# Thyroidectomy in dogs with thyroid tumors: Survival analysis in 144 cases (1994‐2018)

**DOI:** 10.1111/jvim.16644

**Published:** 2023-02-27

**Authors:** Daniela Enache, Livia Ferro, Emanuela M. Morello, Federico Massari, Giorgio Romanelli, Stefano Nicoli, Stefano Guazzetti, Federico Porporato, Eric Zini

**Affiliations:** ^1^ AniCura Istituto Veterinario Novara Granozzo con Monticello (NO) Italy; ^2^ Dipartimento di Scienze Veterinarie University of Turin Grugliasco (TO) Italy; ^3^ Clinica Veterinaria Nervianese Nerviano (MI) Italy; ^4^ Centro Specialistico Veterinario Milan Italy; ^5^ Azienda Unità Sanitaria Locale—IRCCS di Reggio Emilia Reggio Emilia Italy; ^6^ Clinic for Small Animal Internal Medicine, Vetsuisse Faculty University of Zurich Zurich Switzerland; ^7^ Department of Animal Medicine, Production and Health University of Padova Legnaro (PD) Italy

**Keywords:** canine, carcinoma, competing risks analysis, follicular, metastasis, surgery

## Abstract

**Background:**

Few studies have assessed predictors of outcome in dogs with thyroid tumors undergoing thyroidectomy.

**Objective:**

To estimate the survival and identify prognostic factors in dogs with thyroid tumors treated by thyroidectomy.

**Animals:**

A total of 144 client‐owned dogs with thyroid neoplasia that underwent thyroidectomy.

**Methods:**

Retrospective study. Data for analysis included hospital attended and year of surgery, signalment, thyroxine concentration, thyroid tumor features (lobe involvement, size, invasiveness, histopathological type), thrombosis, metastasis, additional surgery and therapy, administration of adjuvant chemotherapy. The association of predictors with survival (time from surgery to death) were assessed by calculating cause‐specific hazard ratios (HR_cs_) and 95% confidence intervals (CI). Causes of death were classified as thyroid‐related or because of other cause.

**Results:**

Overall median survival time was 802 days (CI95% = 723‐1015 days); 89 dogs (77.4%) survived >500 days. Metastases were identified at admission in 12 (8.3%) dogs and were associated with higher thyroid cancer‐related fatality (HR = 5.83, CI95% = 1.56‐21.78; *P* = .009). Thrombosis occurred in 40 dogs and was associated with increased risk of death because of other cause (HR = 2.73, CI95% = 1.18‐6.35; *P* = .019). Nonfollicular carcinoma (HR = 4.17, CI95% = 1.27‐13.69; *P* = .018) and administration of chemotherapy (HR = 3.45, CI95% = 1.35‐8.82; *P* = .01) were associated with higher risk of thyroid cancer‐related death.

**Conclusions and Clinical Importance:**

Dogs with thyroid tumors undergoing thyroidectomy have a long life expectancy. Despite the rare presence of nonfollicular carcinoma and metastases, thyroidectomy should still be considered in some of these dogs.

**Abbreviations**
CI: confidence interval
CIF: cumulative incidence function
HR: hazard ratio
HR_OC_
: hazard ratio for other cause of death
HR_TC_
: hazard ratio for thyroid cancer
MST: median survival time
OR: odd ratio

## 1 INTRODUCTION

Thyroid tumors in dogs have a prevalence of 1‐4% among all neoplasms, and represent the most common tumor of the neuroendocrine system.^1^, ^2^, ^3^, ^4^, ^5^ Older dogs are affected more frequently,^6^, ^7^ and the Beagle, Boxer and Golden Retriever breeds are overrepresented.^2^, ^3^, ^6^, ^8^ Unilateral thyroid lobe involvement is more common than bilateral,^6^, ^9^, ^10^ and approximately 50‐60% of affected dogs are euthyroid, 30‐40% are hypothyroid, and 10‐20% are hyperthyroid.^5^

Carcinomas are the most frequent thyroid tumors in dogs and, among them, 70% arise from follicular cells (ie, follicular thyroid carcinomas) and 30% from parafollicular cells (ie, medullary thyroid carcinomas), both are invasive. Adenomas, on the other hand, are rare in dogs.^3^, ^4^, ^5^, ^11^, ^12^ In dogs, follicular thyroid carcinomas are histologically classified as well‐differentiated (ie, follicular, compact, follicular‐compact and papillary), poorly differentiated, undifferentiated, and carcinosarcoma (malignant mixed thyroid tumor).^12^

At diagnosis, 16‐38% of dogs with thyroid tumors have distant metastasis.^4^, ^13^ Thyroidectomy represents the treatment of choice for freely movable tumors and sometimes could be indicated also for nonresectable ones, along with external beam radiation or radionuclide therapy, chemotherapy or a combination of these therapies.^10^, ^14^, ^15^

There are many purported factors potentially associated with less favorable outcome. Undifferentiated carcinomas have a less favorable outcome,^5^, ^16^, ^17^ however there are conflicting reports in the literature regarding local invasiveness and outcome.^7^, ^18^, ^19^ Tumor size was a negative prognostic factor that appeared also related to the presence of metastases, in an investigation with specimens obtained at necropsy,^20^ but not in other reports with measurements performed at the time of thyroidectomy.^14^, ^16^, ^18^ Thrombosis of the tumor and cervical vessels negatively affects the outcome.^7^ Higher WHO staging and metastatic disease (local or distant) might not result in a poor outcome.^14^, ^21^

A median survival time (MST) of 500‐1100 days is reported after thyroidectomy.^7^, ^18^, ^19^, ^22^, ^23^ In particular, MST >960 days is documented both in dogs undergoing unilateral and bilateral thyroidectomy,^18^, ^23^ and 630 days in dogs with gross vascular invasion treated with unilateral or bilateral thyroidectomy.^19^ Bilateral thyroidectomy negatively affects outcome and the MST of these dogs is 365 days.^14^ Of dogs with functional tumors undergoing thyroidectomy the MST is 2190 days.^24^

No benefit of adjuvant chemotherapy on MST is observed in dogs treated with thyroidectomy alone vs thyroidectomy combined with chemotherapy, irrespective of metastasis.^14^ Administration of toceranib phosphate on macroscopic disease to affected dogs, for a minimum of 10 weeks is beneficial in 75% of cases (ie, complete response to stable disease).^25^ Conversely, no benefit on MST is observed in dogs treated with thyroidectomy alone vs thyroidectomy combined with chemotherapy, irrespective of metastasis.^14^

The aims of the present study were to retrospectively assess survival in dogs with thyroid tumors treated by thyroidectomy, and to identify prognostic factors using competing risk analysis.

## 2 MATERIALS AND METHODS

### 2.1 Experimental design

This is a multi‐institutional retrospective case series study.

### 2.2 Case selection

Medical records of 4 of the authors' institutions (EMM, FM, GR, SN) were retrospectively reviewed in order to identify dogs diagnosed with thyroid tumors that had undergone thyroidectomy between July 18, 1994 and June 20, 2018. Thyroidectomies were performed by both boarded (EMM, FM, GR) and specialized surgeons (SN). Dogs were included if the thyroidectomy had been performed by 1 of the above authors and if follow‐up was available.

For each dog included in the study, variables retrieved from medical records were collected and used to determine their influence on MST, defined as the time from thyroidectomy to death or administrative censoring (last visit). These variables were: the institution where thyroidectomy was performed and the year of surgery, the age (in years, at the time of surgery), gender, neutering status (ie, intact, spayed and castrated), breed, dog size (ie, small, <10 kg; medium, 10‐25 kg; large, >25 kg), presurgical serum thyroxine (T4) concentration (ie, increased, normal or decreased), thyroid lobe involvement (ie, unilateral or bilateral), type of thyroidectomy (ie, unilateral or bilateral), additional surgeries performed (ie, cervical lymphadenectomy and muscular, internal jugular vein resection and parathyroid gland autotransplantation^26^), tumor major axis size, histopathological tumor type, local invasiveness (determined by palpation characteristics of the ventral cervical area combined with computed tomography examination of the same area in some dogs and histopathological examination in others, or histopathological examination in others), thrombosis, metastatic disease at the time of surgery (determined by computer tomography and histopathological examination), administration of adjuvant chemotherapy, radiation therapy after thyroidectomy, levothyroxine and calcitriol supplementation following surgery. In dogs diagnosed with bilateral thyroid lobes tumor involvement, who undergone bilateral thyroidectomy, at least 1 of the parathyroid glands was transplanted. The parathyroid gland was freed from his thyroid capsule, inserted in a muscular pocket, which allowed revascularization and regaining functionality after few weeks. The effect of each aforementioned variable on survival was studied in relation to the cause of death which was eithers thyroid cancer or other cause.

### 2.3 Statistical analysis

Competing risk analysis was used in this study. This was because the classical Kaplan‐Meier analysis, which censors the observed survival time when a competing event intervenes, violates the assumption of independence between time to event and censoring, yielding an underestimate of the survival probability^27^ and overestimating the probability of death. Two competing events were considered, that is, death because of thyroid cancer or death because of another cause. The (cause specific) cumulative incidence function (CIF) estimates the marginal probability for each competing event (ie, the probability of experiencing the event of interest before time t and before the occurrence of a different type of event) as a function of time and was used to estimate the likelihood of the occurrence of each of the 2 causes of death. Gray's test was used to compare cause‐specific CIFs in dogs classified according to each variable gathered at surgery.^28^ The extended Cox proportional hazards model was used to estimate the cause‐specific hazard ratio (HR) for each variable.^29^, ^30^ The model accounted for the multicentric nature of the study by allowing different baseline hazards for the 4 clinics. The proportional hazards assumption was evaluated by studying the Schoenfeld residuals.^31^ Linearity was assumed based on the analysis of the martingale residuals. After an initial assessment, the effect of neutering was not considered.

In order to preserve the sample size, the random survival forest algorithm for imputing missing data was used.^32^ Bivariate associations between variables were investigated by Fisher's exact test and *t*‐test. Logistic regression was used to model the probability of concurrent metastasis and thrombosis at diagnosis, and odds ratios (ORs) were calculated. All analyses were performed using R with *mstate*, *cmprsk*, *randomForestSRC* and *survival* packages.^31^, ^33^, ^34^, ^35^

## 3 RESULTS

### 3.1 Dogs

Medical records of 144 dogs with thyroid tumors that had undergone thyroidectomy fulfilled the inclusion criteria and were considered for analysis. Dogs were treated in the 4 authors' institutions, named A, B, C and D; of which, 78 (54.2%) were treated in A, 24 (16.7%) in B, 34 (22.9%) in C and 9 (6.3%) in D. Seventy‐four dogs (51.4%) were female and 66 (45.8%) were male; gender was not available in 4 cases (2.8%). Among females, 39 (52.7%) were intact and 35 (47.3%) were spayed, and among males 16 (24.2%) were intact and 50 (75.8%) were neutered. Median age at the time of surgery was 10 years (range: 4‐15). Forty‐two (29.2%) dogs were crossbreed and 102 (70.8%) were purebred. Beagles (n = 11) and Boxers (n = 11) were most represented, followed by Golden Retrievers (n = 10), English Setters (n = 10) and Siberian Huskies (n = 7). Of the 144 dogs, 13 (9%) were small (<10 kg), 17 (11.8%) medium (10‐25 kg), and 73 (50.7%) large (>25 kg); size was unknown in 41 cases (28.5%).

The affected thyroid lobe was recorded in 135 (93.8%) dogs; 122 (91.1%) cases had unilateral and 13 (9%) had bilateral involvement. Presurgical serum T4 concentration was measured in 103 (71.5%) dogs; 9 (8.7%) cases had abnormally high T4, all with unilateral involvement, 7 (6.8%) cases had abnormally low T4, including 6 cases with unilateral and 1 with bilateral involvement, and 87 (84.5%) cases were euthyroid. Of the euthyroid cases, thyroidal lobe involvement was recorded in 52 (59.8%), including 45 (86.5%) with unilateral, and 7 (13.5%) with bilateral involvement.

The year of surgery was available for all dogs; surgery was performed from July 18, 1994 to June 20, 2018 (Table 1). Thyroidectomy alone was performed in 109 of the 144 (75%) dogs, while in 35 (24.3%) additional surgery was required, including cervical lymphadenectomy, muscular and internal jugular vein resection, and parathyroid gland autotransplantation.^26^ Often, even with marginal resection it was possible to obtain a complete removal of the thyroid tumors, which was considered curative. Whenever possible, the surgical procedures performed have led to complete removal of the thyroid tumors, even with small margins. In those cases where local invasion of the blood vessels or nerves was noted, resection of the venous structures was performed, while the arterial and nervous structures were subjected to debulking with safeguarding of the latter. This approach has still allowed an excellent local control of the thyroid tumors. At surgery, the mean major axis of the excised lobe was measured in 142 (98.6%) dogs which was 4.6 cm (range, 4‐16). Of 141 (97.9%) dogs with histopathologic assessment of the thyroid sample, 128 (90.8%) had differentiated and 13 (9.2%) had undifferentiated thyroid tumors. One (0.8%) of the 129 dogs with a differentiated thyroid tumor had an adenoma, while the remaining 128 (99.2%) had a carcinoma. Computed tomography of the skull, ventral cervical area, thorax and abdomen was performed in 91 (62.8%) dogs; tumor and jugular vein thrombosis was identified in 40 of 144 (27.8%) cases, and lymphadenopathy and suspected metastases in 12 (8.3%). Other dogs included in the study had similar staging tests (ie, thoracic x‐rays, abdominal ultrasound scan). In case of regional lymphadenopathy (eg, prescapular, mandibular, retropharyngeal), a fine needle aspiration was performed in order to evaluate the presence of metastases, while the lung nodules were suspected of being metastases by means of computer tomography images.

**TABLE 1:** Descriptive statistics of dogs with thyroid tumors treated by thyroidectomy.

	Surviving (N = 55)	Death by thyroid cancer (N = 29)	Death by other cause (N = 60)	Overall (N = 144)
Sex				
Female	27 (49.1%)	18 (62.1%)	29 (48.3%)	74 (51.4%)
Male	26 (47.3%)	10 (34.5%)	30 (50%)	66 (45.8%)
Not available	2 (3.6%)	1 (3.4%)	1 (1.7%)	4 (2.8%)
Age (years)				
Mean (SD)	9.97 (2.33)	9.11 (2.47)	9.81 (2.43)	9.73 (2.41)
Median [Min, Max]	11 [4, 15]	9 [5, 13]	10 [5, 14]	10 [4, 15]
Not available	2 (3.6%)	1 (3.4%)	1 (1.7%)	4 (2.8%)
Breed				
Crossbreed	13 (23.6%)	13 (44.8%)	16 (26.7%)	42 (29.2%)
Purebred	42 (76.4%)	16 (55.2%)	44 (73.3%)	102 (70.8%)
Size				
Small	8 (14.5%)	2 (6.9%)	3 (5%)	13 (9%)
Medium	8 (14.5%)	1 (3.4%)	8 (13.3%)	17 (11.8%)
Large	26 (47.3%)	14 (48.3%)	33 (55%)	73 (50.7%)
Not available	13 (23.6%)	12 (41.4%)	16 (26.7%)	41 (28.5%)
T4 levels				
Normal	30 (54.5%)	17 (58.6%)	40 (66.7%)	87 (60.4%)
Increased	5 (9.1%)	2 (6.9%)	2 (3.3%)	9 (6.3%)
Decreased	2 (3.6%)	1 (3.4%)	4 (6.7%)	7 (4.9%)
Not available	18 (32.7%)	9 (31%)	14 (23.3%)	41 (28.5%)
Year of surgery				
1994	0 (0%)	2 (6.9%)	2 (3.3%)	4 (2.8%)
1997	1 (1.8%)	0 (0%)	2 (3.3%)	3 (2.1%)
1998	1 (1.8%)	1 (3.4%)	1 (1.7%)	3 (2.1%)
1999	2 (3.6%)	0 (0%)	1 (1.7%)	3 (2.1%)
2000	0 (0%)	0 (0%)	2 (3.3%)	2 (1.4%)
2001	2 (3.6%)	2 (6.9%)	1 (1.7%)	5 (3.5%)
2002	1 (1.8%)	0 (0%)	4 (6.7%)	5 (3.5%)
2003	1 (1.8%)	1 (3.4%)	3 (5%)	5 (3.5%)
2004	0 (0%)	0 (0%)	2 (3.3%)	2 (1.4%)
2005	0 (0%)	2 (6.9%)	5 (8.3%)	7 (4.9%)
2006	2 (3.6%)	3 (10.3%)	1 (1.7%)	6 (4.2%)
2007	0 (0%)	0 (0%)	4 (6.7%)	4 (2.8%)
2008	1 (1.8%)	1 (3.4%)	7 (11.7%)	9 (6.3%)
2009	2 (3.6%)	5 (17.2%)	4 (6.7%)	11 (7.6%)
2010	1 (1.8%)	2 (6.9%)	5 (8.3%)	8 (5.6%)
2011	3 (5.5%)	2 (6.9%)	3 (5%)	8 (5.6%)
2012	3 (5.5%)	0 (0%)	3 (5%)	6 (4.2%)
2013	3 (5.5%)	2 (6.9%)	3 (5%)	8 (5.6%)
2014	6 (10.9%)	2 (6.9%)	3 (5%)	11 (7.6%)
2015	6 (10.9%)	1 (3.4%)	3 (5%)	10 (6.9%)
2016	4 (7.3%)	3 (10.3%)	0 (0%)	7 (4.9%)
2017	10 (18.2%)	0 (0%)	0 (0%)	10 (6.9%)
2018	6 (10.9%)	0 (0%)	1 (1.7%)	7 (4.9%)
Major axis (cm)				
Mean (SD)	4.65 (2.8)	6.26 (2.88)	5.33 (2.18)	5.25 (2.63)
Median [min, max]	4 [1, 16]	6 [2.5, 13]	5 [2, 12]	4.75 [1, 16]
Not available	0 (0%)	0 (0%)	2 (3.3%)	2 (1.4%)
Histology				
Undifferentiated	3 (5.5%)	7 (24.1%)	3 (5%)	13 (9%)
Follicular	50 (90.9%)	22 (75.9%)	56 (93.3%)	128 (88.9%)
Not available	2 (3.6%)	0 (0%)	1 (1.7%)	3 (2.1%)
Lobar involvement				
Unilateral	46 (83.6%)	29 (100%)	47 (78.3%)	122 (84.7%)
Bilateral	4 (7.3%)	0 (0%)	9 (15%)	13 (9%)
Not available	5 (9.1%)	0 (0%)	4 (6.7%)	9 (6.3%)
Metastasis				
No	52 (94.5%)	21 (72.4%)	59 (98.3%)	132 (91.7%)
Yes	3 (5.5%)	8 (27.6%)	1 (1.7%)	12 (8.3%)
Thrombosis				
No	43 (78.2%)	17 (58.6%)	44 (73.3%)	104 (72.2%)
Yes	12 (21.8%)	12 (41.4%)	16 (26.7%)	40 (27.8%)
Additional surgery				
No	45 (81.8%)	17 (58.6%)	47 (78.3%)	109 (75.7%)
Yes	10 (18.2%)	12 (41.4%)	13 (21.7%)	35 (24.3%)
Chemotherapy				
No	43 (78.2%)	11 (37.9%)	43 (71.7%)	97 (67.4%)
Yes	11 (20%)	16 (55.2%)	13 (21.7%)	40 (27.8%)
Not available	1 (1.8%)	2 (6.9%)	4 (6.7%)	7 (4.9%)
Levothyroxine				
No	46 (83.6%)	28 (96.6%)	50 (83.3%)	124 (86.1%)
Yes	8 (14.5%)	1 (3.4%)	9 (15%)	18 (12.5%)
Not available	1 (1.8%)	0 (0%)	1 (1.7%)	2 (1.4%)
Calcium or vitamin D				
No	49 (89.1%)	28 (96.6%)	51 (85%)	128 (88.9%)
Yes	5 (9.1%)	1 (3.4%)	8 (13.3%)	14 (9.7%)
Not available	1 (1.8%)	0 (0%)	1 (1.7%)	2 (1.4%)
Center				
A	27 (49.1%)	14 (48.3%)	37 (61.7%)	78 (54.2%)
B	15 (27.3%)	6 (20.7%)	3 (5%)	24 (16.7%)
C	8 (14.5%)	8 (27.6%)	17 (28.3%)	33 (22.9%)
D	5 (9.1%)	1 (3.4%)	3 (5%)	9 (6.3%)

Among all dogs, 40 (27.6%) received adjuvant chemotherapy (Table 1); in 7 out of 144 (4.8%), the information regarding the use of adjuvant chemotherapy was missing. Radiotherapy (once, unknown dose) and doxorubicin (30 mg/m^2^, q3w, 3 times) was administered in 1 dog, cyclophosphamide (15 mg/m^2^, q24h, metronomic) and doxorubicin (1 mg/kg, q3w) in 1 dog, toceranib (2.8 mg/kg, 3 times/week, 120 times) in 1 dog, cyclophosphamide (10 mg/m^2^, q24h, metronomic) in 1 dog, carboplatin (300 mg/m^2^, q3w, 4 times) in 8 dogs, doxorubicin (1 mg/kg, q3w, 2 times) in 2 dogs, doxorubicin (1 mg/kg, q3w, 3 times) in 4 dogs, doxorubicin (1 mg/kg, q3w, 5 times) in 6 dogs, while the remaining dogs have received doxorubicin (1 mg/kg, q3w, 4 times).

Eighteen (12.4%) dogs received levothyroxine supplementation and 14 (9.7%) calcitriol and calcium following surgery, or calcium following surgery. Of the latter, 12 (85.7%) cases had bilateral thyroid lobes involvement and benefited also from autotransplantation of 1 of the parathyroid glands. Twelve (66.7%) of these 18 dogs were euthyroid before thyroidectomy, 2 (11.1%) had decreased T4, 2 (11.1%) had increased T4, and in the remaining 2 (11.1%) the thyroid status was not recorded before surgery.

### 3.2 Risk of death during study period

**FIGURE 1:**
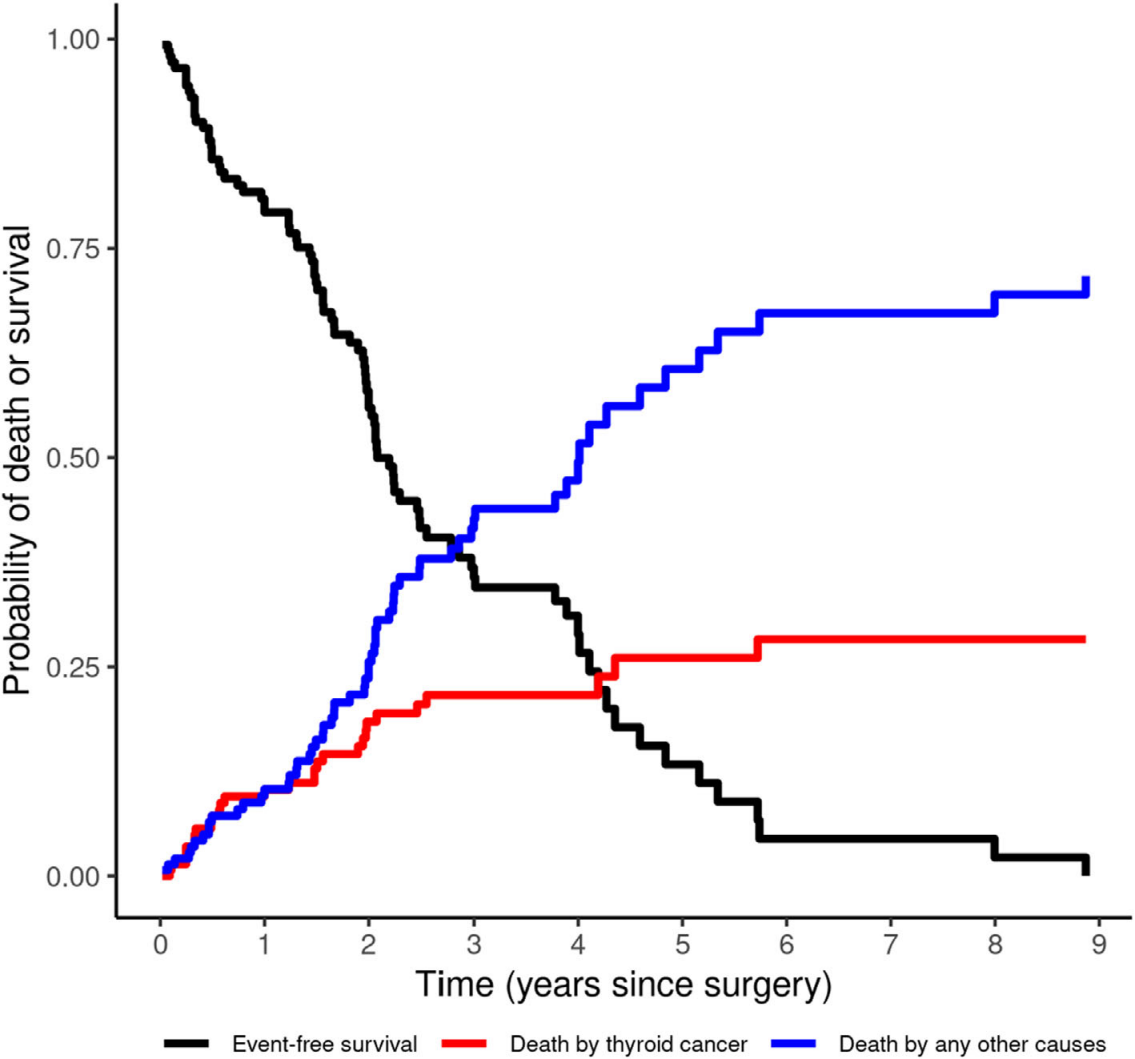
Kaplan‐Meier estimate of overall survival (ie, regardless of the death cause) and cumulative incidence (mortality) functions for each of the 2 outcomes (death by thyroid cancer and death by other cause).

The observational period ranged between 7 and 3240 days (median 610 days), with the accrual of 280 dog‐years. During this interval, 29 (20.1%) dogs died because of thyroid cancer and 60 (41.7%) died because of other causes (laryngeal paralysis in 1 dog, dog fight in 2 dogs, pulmonary thromboembolism in 1 dog, hepatocellular carcinoma with metastasis in 1 dog, and euthanasia because of unrelated thyroid cancer diseases, hypothesized based on clinical records in the remaining dogs); necropsies were not performed, and the causes of death were inferred from the clinical records. The remaining 55 (38.2%) dogs were still alive at the time of data collection or were lost to follow‐up and their survival time was right censored at the time of the last examination. The median time to censoring was 581 (minimum 30, maximum 1453). Cumulative mortality functions for both causes of death (because of thyroid cancer or other cause) and overall (ie, for all the causes) survival of dogs are reported in Figure 1. The MST for the 144 dogs was 802 days (CI95% = 723‐1045); of which, 89 (74.04%) survived >500 days. The MST and cumulative mortality function for both causes of death is detailed in Table 2. The probability of dying because of thyroid cancer was estimated to be 10.4% after 1 year, whereas the probability of dying from other cause was 10.8%. Two years after surgery, the probability of dying because of thyroid cancer was 18.6%, which after 5 years increased to approximately 26%, leveling off to 28.6% after 6 years. The risk of death for other causes continued to increase over time.

**TABLE 2:** Cumulative incidence (mortality) and Kaplan‐Meier survival at various time points in dogs with thyroid tumor treated by thyroidectomy.

	Cumulative mortality (%)		
	Thyroid cancer	Other cause	Overall survival
One month	0.3	1.4	98.3
Two months	1.9	2.1	96.0
Three months	3.5	2.1	94.4
Six months	7.2	7.3	85.5
One year	10.4	10.5	79.2
Two years	18.6	25.9	55.5
Three years	21.8	42.3	35.9
Four years	21.8	49.4	28.8
Five years	26.3	61.3	12.4
Six years	28.6	67.2	4.2
Seven years	28.6	68.2	3.2
Eight years	28.6	69.2	2.2

### 3.3 Cause‐specific CIF

**FIGURE 2:**
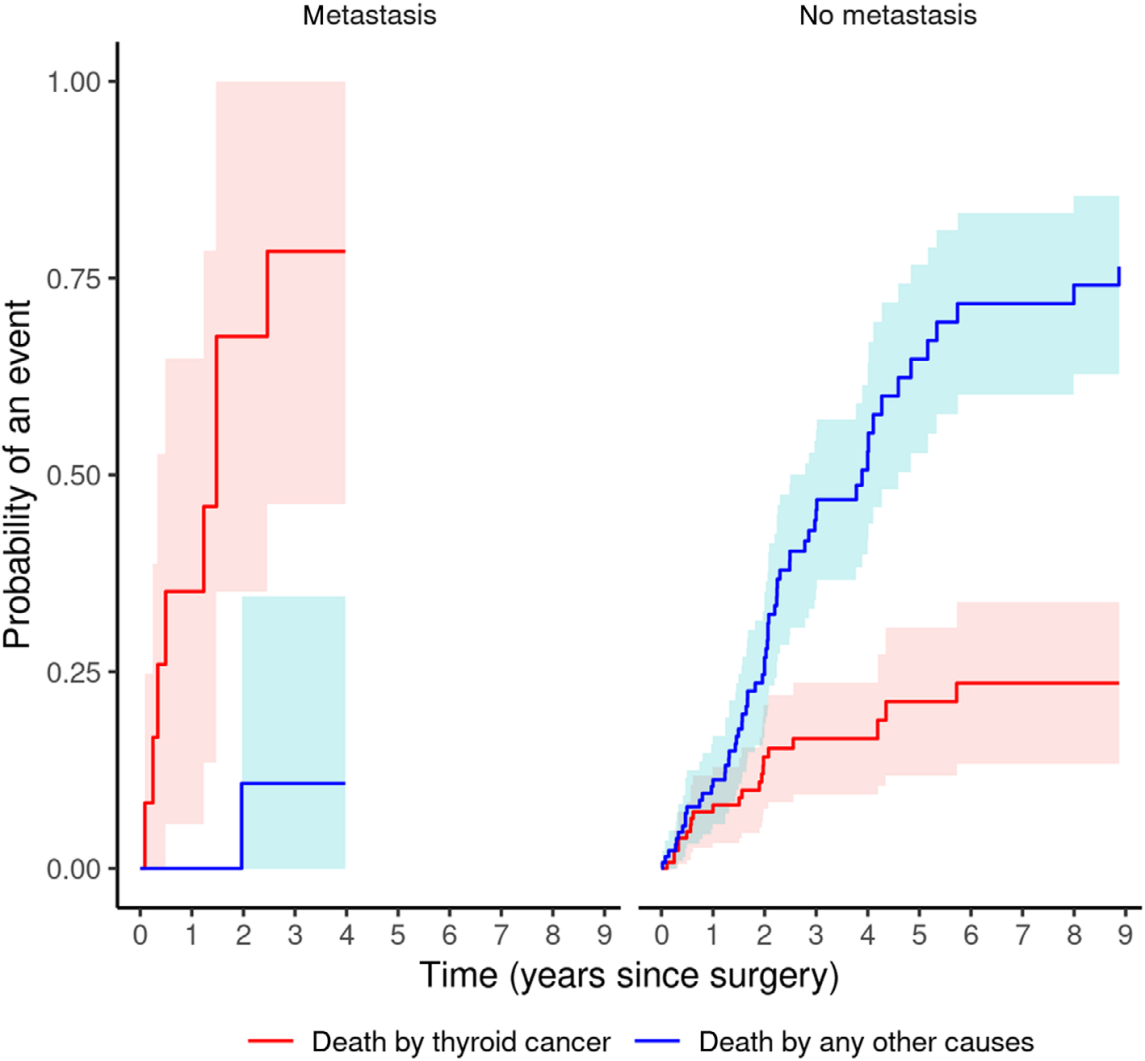
CIFs for each of the 2 outcomes for subjects with or without metastasis at the time of surgery.

The estimated effect of metastasis on cause‐specific CIFs is shown in Figure 2 and Table 3. A higher risk of death because of thyroid cancer was observed in dogs with metastasis (CIF = 0.00 at 30 days, CIF = 0.35 at 365 days; *P* < .001), while the presence of metastasis did not affect risk of death because of other causes of death. No dog with metastasis survived more than 2 years and a half.

**TABLE 3:** Cause‐specific cumulative mortality by presence or absence of metastasis, at various time points after surgery, [CIF].

	Death by other cause		Death by thyroid cancer	
Years	No metastasis	Metastasis	No metastasis	Metastasis
1	0.11	0.00	0.08	0.35
2	0.27	0.11	0.14	0.68
3	0.45	0.11	0.17	0.78
4	0.52		0.17	
5	0.64		0.21	
6	0.71		0.24	
7	0.71		0.24	
8	0.74		0.24	

**FIGURE 3:**
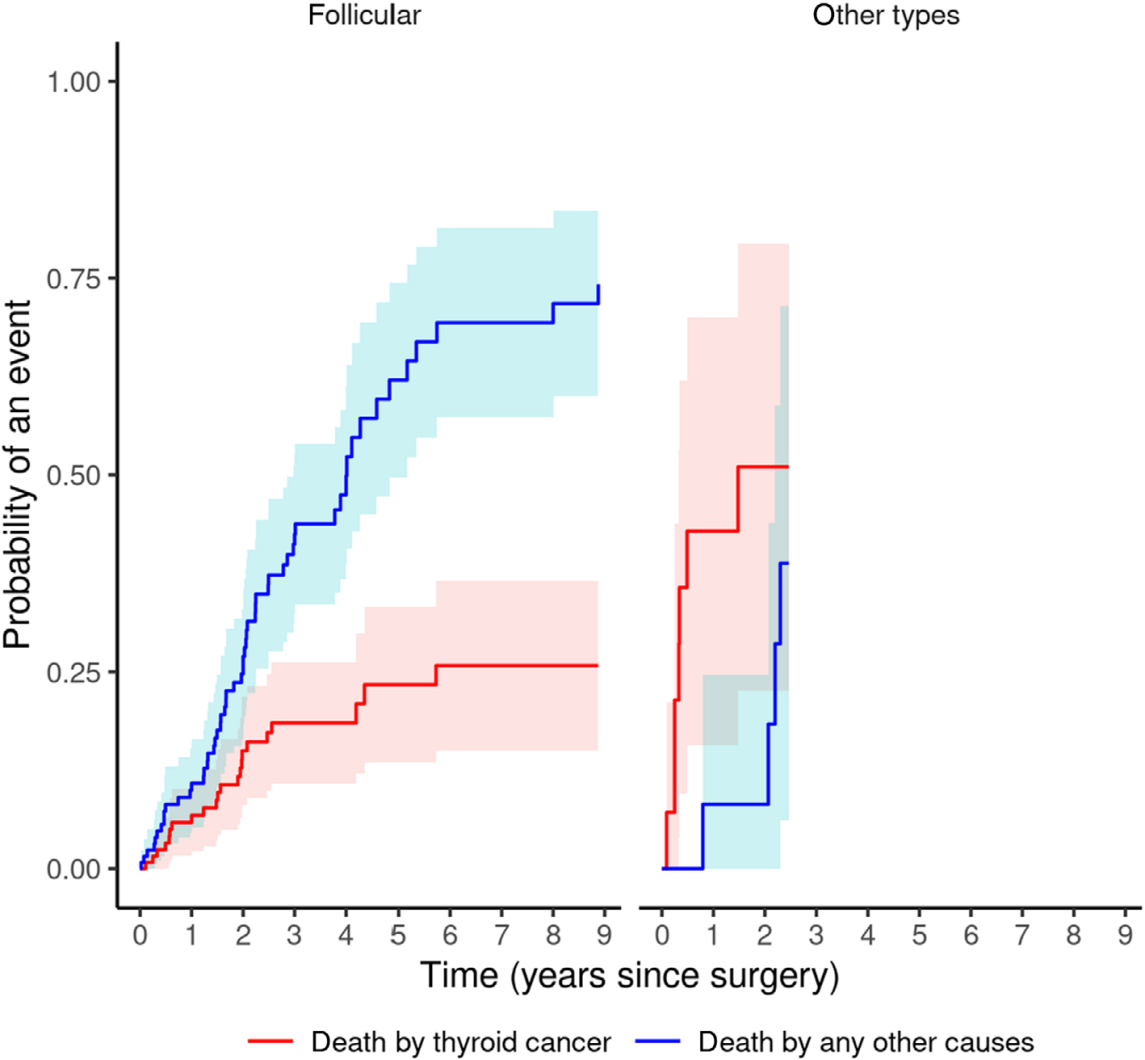
CIFs for each of the 2 outcomes for subjects with follicular or nonfollicular thyroid cancer.

Regarding histological type, prognosis in dogs with thyroid carcinoma with undifferentiated type (CIF = 0.00 at 30 days, CIF = 0.46 at 365 days) was worse than in those with follicular type (CIF = 0.00 at 30 days, CIF = 0.09 at 365 days; *P* < .001). The histological type did not affect risk of death because of other cause (Figure 3, Table 4).

**TABLE 4:** Cause‐specific cumulative mortality by histological type, at various time points after surgery.

	Death by other cause		Death by thyroid cancer	
Years	Follicular	Other types	Follicular	Other types
1	0.11	0.09	0.07	0.46
2	0.27	0.09	0.15	0.55
3	0.42		0.19	
4	0.5		0.19	
5	0.62		0.23	
6	0.69		0.26	
7	0.69		0.26	
8	0.72		0.26	

**FIGURE 4:**
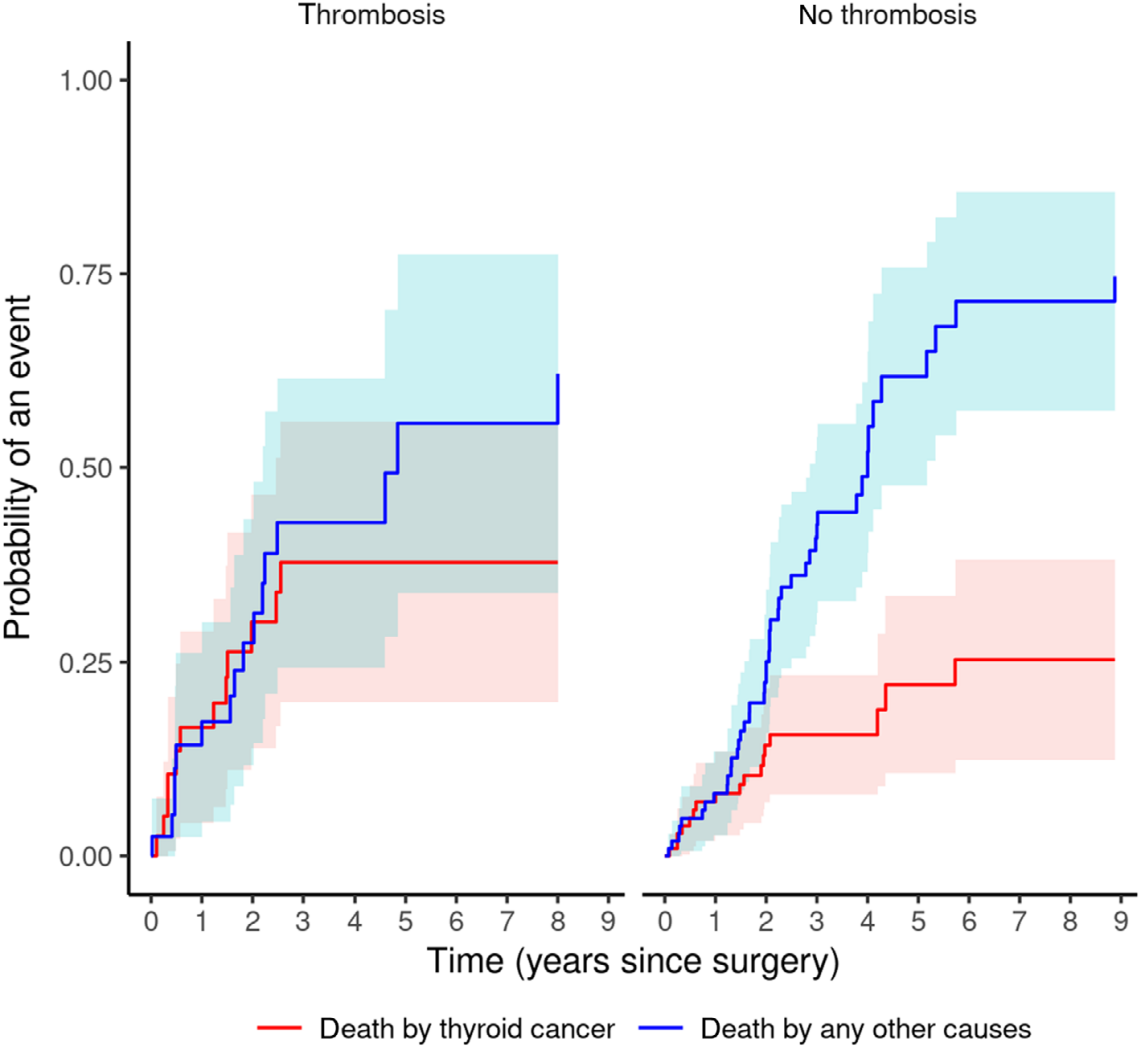
CIFs for each of the 2 outcomes for subjects with or without thrombosis at the time of surgery.

The presence of thrombosis was associated with a higher risk of death because of other causes (CIF = 0.03 at 30 days, CIF = 0.62 at 2000 days; *P* = .044), but did not affect risk of death because of thyroid tumor (Figure 4, Table 5).

**TABLE 5:** Cause‐specific cumulative mortality by presence or absence of thrombosis, at various time points after surgery.

	Death by other cause		Death by thyroid cancer	
Years	No thrombosis	Thrombosis	No thrombosis	Thrombosis
1	0.08	0.17	0.08	0.17
2	0.25	0.27	0.14	0.3
3	0.42	0.43	0.16	0.38
4	0.51	0.43	0.16	0.38
5	0.61	0.56	0.22	0.38
6	0.71	0.56	0.26	0.38
7	0.71	0.56	0.26	0.38
8	0.71	0.62	0.26	0.38

**FIGURE 5:**
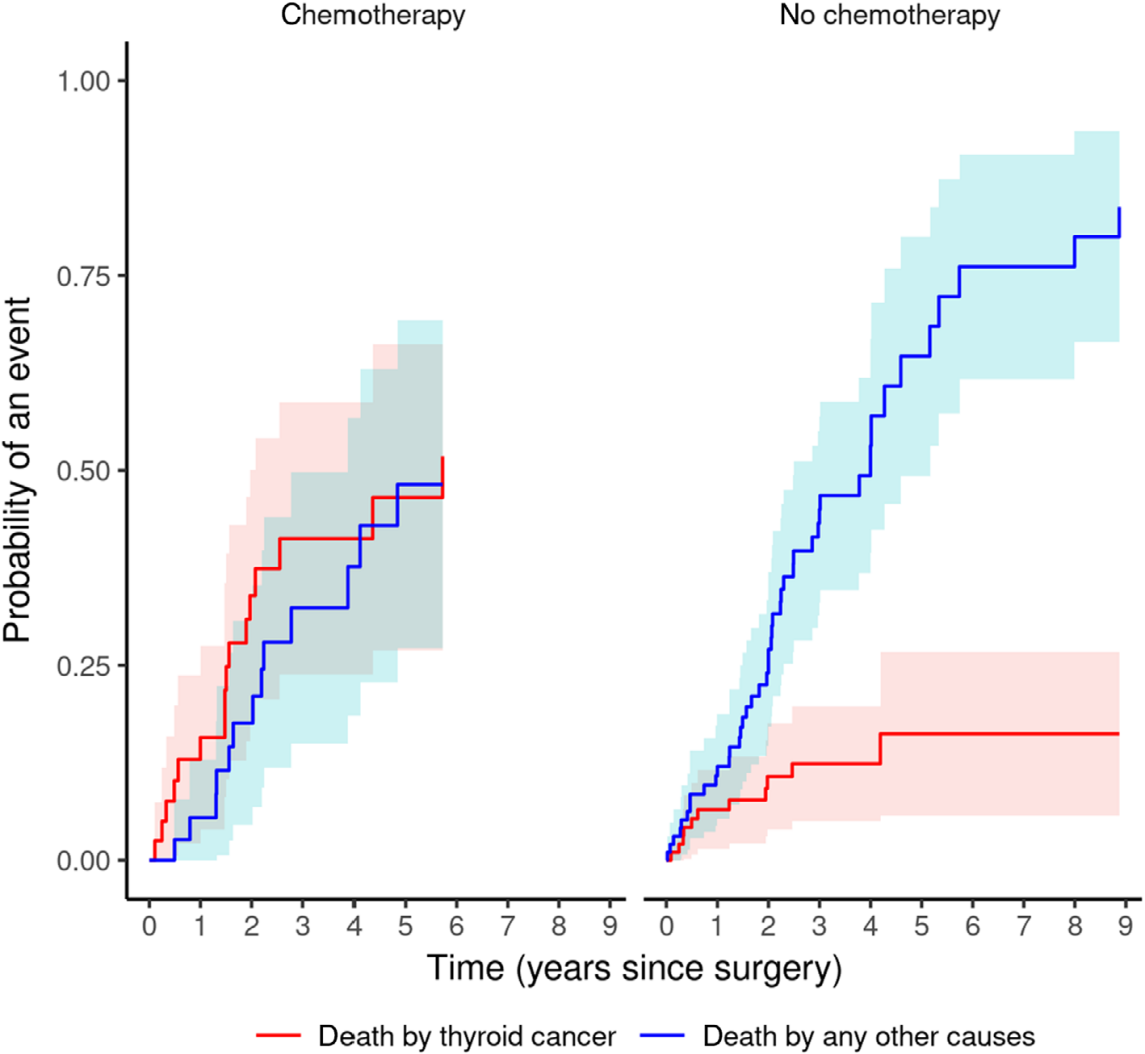
CIFs for each of the 2 outcomes for subjects with or without postoperative chemotherapy.

Administration of adjuvant chemotherapy was associated with an increase in risk of death because of thyroid cancer (CIF = 0.00 at 30 days, CIF = 0.16 at 365 days; *P* < .001) but not because of other causes (Figure 5, Table 6).

**TABLE 6:** Cause‐specific cumulative mortality by administration of postoperative chemotherapy, at various time points after surgery.

	Death by other cause		Death by thyroid cancer	
Years	No chemotherapy	Chemotherapy	No chemotherapy	Chemotherapy
1	0.12	0.05	0.07	0.16
2	0.27	0.18	0.11	0.34
3	0.44	0.32	0.13	0.41
4	0.52	0.38	0.13	0.41
5	0.64	0.48	0.16	0.47
6	0.76		0.16	
7	0.76		0.16	
8	0.8		0.16	

### 3.4 Cause‐specific hazard ratios

The cause‐specific hazard ratios for thyroid cancer (HR_TC_) and for other cause of death (HR_OC_) are shown in Table 7 and represent the adjusted effect of the covariates on the hazard of the specific event. As expected, older age was associated with death because of other cause (HR_OC_ for an increase of 1 year of age = 1.39, CI95% = 1.19‐1.61; *P* < .001); older age was not associated with death because of thyroid cancer. Neither sex nor breed were associated with the risk of death because of thyroid cancer and other cause. In addition, the risk of death because of other cause was higher for large sizes compared to small size dogs (HR_OC_ = 4.3, CI95% = 1.05‐18; *P* = .043); no association was found with thyroid cancer‐related death. Serum T4 concentration, tumor size, additional surgery, administration of levothyroxine, calcium and calcitriol were not associated with any causes of death.

**TABLE 7:** Cause‐specific hazard ratios and 95% confidence interval for each covariate based on the extended Cox Proportional Hazard model calculated for the 2 competing causes of death.

	Thyroid cancer				Other cause			
	HR	HR 95% lower limit	HR 95% upper limit	*P* value	HR	HR 95% lower limit	HR 95% upper limit	*P* value
Age	0.944	0.767	1.162	.587	1.383	1.188	1.611	<.001
Sex (male vs female)	0.573	0.23	1.427	.231	0.926	0.495	1.729	.808
Breed (pure vs mixed)	1.04	0.383	2.826	.939	1.59	0.782	3.235	.2
Size (medium vs small)	0.341	0.027	4.333	.407	4.483	0.864	23.259	.074
Size (large vs small)	0.262	0.04	1.713	.162	4.34	1.047	17.992	.043
Histology (undifferentiated vs follicular)	4.175	1.273	13.689	.018	1.964	0.496	7.775	.336
T4 increased (vs normal)	0.401	0.072	2.231	.297	0.574	0.112	2.949	.507
T4 decreased (vs normal)	1.353	0.141	12.987	.794	0.888	0.213	3.701	.871
Major axis (cm)	1.148	0.964	1.366	.122	1.036	0.906	1.185	.605
Thrombosis (yes vs no)	2.144	0.735	6.25	.163	2.732	1.178	6.336	.019
Metastasis (yes vs no)	5.831	1.561	21.778	.009	0.507	0.055	4.653	.548
Levothyroxine (yes vs no)	0.347	0.038	3.179	.349	1.366	0.533	3.504	.516
Additional surgery (yes vs no)	0.593	0.19	1.857	.37	0.868	0.308	2.447	.79
Chemotherapy (yes vs no)	3.448	1.347	8.821	.01	0.383	0.145	1.015	.053
Year of surgery (years)	0.952	0.855	1.059	.363	0.917	0.857	0.981	.012

Histological tumor type was associated with the probability of dying because of thyroid cancer but not to other cause. Notably, dogs with nonfollicular tumors had an approximate 4‐fold higher risk of death because of thyroid cancer than those with the follicular type (HR_TC_ = 4.18, CI95% = 1.27‐13.69; *P* = .018).

Thrombosis was associated with a higher risk of death because of other causes: pulmonary thromboembolism in 2 dogs, acute kidney injury in 1 dog, cardiac tamponade in 1 dog, congestive heart failure in 1 dog, frontal sinus carcinoma in 1 dog, mediastinal tumor in 1 dog, splenic hemangiosarcoma in 1 dog, gastric dilatation and volvulus in 1 dog, cardiac base tumor in 1 dog, lung disease not caused by neoplasia in 1 dog, transitional cell carcinoma in 1 dog, euthanasia because of aggressiveness in 1 dog; the remaining dogs were euthanized because of diseases unrelated to thyroid carcinoma (HR_OC_ = 2.73, CI95% = 1.18‐6.33; *P* = .019) but not to thyroid cancer.

Administration of adjuvant chemotherapy was associated with a higher risk of death because of thyroid cancer (HR_TC_ = 3.45, CI95% = 1.35‐8.82; *P* = .01) but not to other cause; overall MST of dogs treated with adjuvant chemotherapy was shorter than the overall MST of dogs that did not receive adjuvant chemotherapy (710 days, CI95% = 550‐1015; 910 days, CI95% =760‐1465; but the difference was not significant at the logrank test *P* = .064).

The year of surgery was associated with better survival because of other cause (HR_OC_ = 0.92 per year, CI95% = 0.86‐0.98; *P* = .012) highlighting a better outcome for the most recent cases.

Metastasis at surgery was associated with death because of thyroid cancer (HR_TC_ = 5.83, CI95% = 1.56‐21.78; *P* = .009) but not with other cause, with a 6‐fold increase in the risk of death by thyroid cancer in dogs compared to those without tumor spread; overall MST of dogs with metastasis was 540 days, and 818 days for dogs without metastasis, 5 out of 12 (41.7%) dogs with tumor spread survived longer than 500 days. Regarding metastasis, associations with other variables were also studied in a multivariate logistic regression model that considered tumor size, histological tumor type, presence of thrombosis and the number of thyroid lobes involved; histological tumor type (OR = 8.1, CI95% = 1.8‐35.2, *P* = .005) was associated with tumor spread.

The presence of metastasis was also associated with that of tumor thrombosis (OR = 3.44, CI95% = 1.07‐16.1; *P* = .045), but not with the other variables.

Tumor thrombosis was observed in 4 cases of the 13 undifferentiated thyroid tumor type and in 8 cases of the 120 follicular thyroid tumor type (*P* = .015).

When modeling the probability of tumor thrombosis in a multivariate logistic regression model, conjointly considering histological type (all other types vs follicular), sex, age, body size, major axis, bilateral thyroid lobe involvement, and metastasis, associations were found with tumor size (OR for 1 cm increase = 1.26, CI95% = 1.09‐1.49; *P* = .003), bilateral thyroid lobe involvement (OR = 4.67, CI95% = 1.4‐17.06; *P* = .014) and presence of metastasis (OR = 3.85, CI95% = 1.02‐15.76; *P* = .048).

## 4 DISCUSSION

We investigated survival in dogs affected by thyroid tumors treated with thyroidectomy. We also examined the outcome predictors by classifying death as thyroid related or because of other cause, using competing risk analysis. The overall (ie, for all causes of death) MST of affected dogs was longer than 2 years. Undifferentiated carcinoma, metastasis and administration of adjuvant chemotherapy were associated with a higher hazard of death because of thyroid tumor. Increasing age, larger body size, presence of thrombosis and less recent surgery were associated with shorter survival time because of other cause.

Regarding the survival of affected dogs, the results of this series are similar to previous investigations that indicated an MST ranging between 500 and 1100 days.^11^, ^19^, ^22^, ^23^, ^36^ Based on the cumulative mortality assessment, in the present study the risk of death because of thyroid cancer during the first year after thyroidectomy was approximately 10%, and at 5 years approximately 25%. Not unexpectedly, the latter was significantly lower than the risk of death because of other cause, which was approximately 60%. Thus, most dogs diagnosed with thyroid tumors that survived the thyroidectomy died because of causes unrelated to thyroid cancer.

With regard to outcome predictors, the present study confirms previous observations that undifferentiated thyroid carcinomas are more aggressive, and have a poorer prognosis.^4^, ^17^, ^36^ In fact, in our study, dogs diagnosed with a nonfollicular carcinoma had a 4‐fold higher risk of death because of thyroid cancer compared to follicular carcinoma. The reported local aggressive behavior of nonfollicular thyroid tumors likely explains the guarded outcome.^7^ Conversely, in some studies the histopathological tumor type did not influence survival in dogs, possibly because of the more limited number of cases included compared to the present investigation.^22^, ^23^, ^37^

In this case series, metastasis at diagnosis was a major risk factor of death in dogs with thyroid cancer, and survival was 35% lower in those with tumor spread. However, although the presence of metastasis at diagnosis was associated with a poor outcome, 6 dogs with metastasis lived longer than 500 days. Hence, thyroidectomy could be taken into consideration in certain cases with metastatic disease, despite the pros and cons that need to be clearly discussed with the owner.

Notably, in a study the presence of metastases in 15 dogs undergoing thyroidectomy did not negatively affect the outcome.^14^ Recently, in an investigation into the prognosis of canine thyroid carcinoma based on computed tomography assisted staging, the presence of metastatic disease had no influence on MST, although a trend toward significance was observed.^21^ The latter study included 58 dogs and given that the whole abdomen was not routinely imaged, the rate of metastatic disease might have been underestimated. Although, in another study it was found that from 15 dogs diagnosed with thyroid carcinoma, 9 dogs had pulmonary metastases, while 1 dog had abdominal metastases spread to the liver and spleen.^38^

In our study, the administration of adjuvant chemotherapy was negatively associated with survival, irrespective of metastasis at surgery, with a hazard of dying of about 3.5 times greater in those that received chemotherapy. It cannot be excluded that this finding was caused by a “confounding by indication” phenomenon, because of the retrospective nature of the study. Although unproven, it is possible that adjuvant chemotherapy was recommended more often and administered to dogs presenting other negative prognostic factors, hence the outcome was poor regardless of chemotherapy. This is supported by the literature, which suggests chemotherapy in dogs presenting with negative prognostic factors, such as metastatic disease, thrombosis, and invasive tumor. In addition, adjuvant chemotherapy could have played a permissive role in some complications, however medical records prevented further clarification. Caution is however required in inferring a causal relationship since chemotherapy was not administered at random but upon clinical indication.

Similar to the present study, adjuvant chemotherapy did not improve survival compared to surgery‐only treated cases.^14^ However, it has also been reported that 30‐50% of dogs treated with doxorubicin or cisplatin had a more than 50% reduction in the thyroid tumor volume,^39^, ^40^ indicating some benefits of chemotherapy on gross disease. Moreover, in dogs with thyroid cancer that had received adjuvant 9‐cis retinoic acid after thyroidectomy, the outcome was better than surgery alone or combined with doxorubicin,^17^ despite the limited number of cases included. In humans diagnosed with differentiated thyroid cancer, systemic cytostatic chemotherapy does not seem to be beneficial for survival.^41^

As expected, in the present series higher age^3^ and body size at the time of surgery were associated with a higher risk of death because of other cause. This is in line with the literature, showing that medium and large‐breed dogs are at increased risk to develop thyroid tumors.^3^, ^6^, ^16^, ^20^ The reasons for this is unclear, but an explanation could be related to the fact that the risk of mutation associated with normal processes of cells replication are enhanced in large breed dogs.^42^

Thrombosis was also identified as a cause of death unrelated to thyroid cancer. In humans with thyroid carcinoma tumor, thrombosis is associated with an increased chance of death because of thyroid cancer but not to all other causes.^43^ The underlying explanation in dogs remains unclear.

In our study, an association between histological tumor type and metastasis was observed. In turn, metastasis was associated with thrombosis, thyroid tumor size, and bilateral tumor lobe involvement. Thus, not unexpectedly, tumors that spread to other sites are frequently those that have the ability to grow larger and invade the vessels.^20^ In addition, similarly to a previous study where larger thyroid tumors were more locally invasive in dogs,^7^ we found that tumor thrombosis was associated with bilateral lobe involvement and a longer tumor major axis. Therefore, it could be suspected that in the pathophysiology of thyroid tumors in dogs, metastasis, thrombosis, and size could be connected, but further investigation is needed in order to verify if this association is consistent.

With regard to the year of surgery, earlier years were associated with an increased risk of death because of other cause. This might be because of improved veterinary care in the last decade, possibly increasing the overall life expectancy of the most recent cases. Moreover, the use of the latest generation surgical instruments allows the closure of the thyroid vascularization in a much safer way in humans. LigaSure, seems to generate less thermal damage to the treated tissues.^44^ In veterinary medicine, their use is associated with a reduction in surgery time but does not seem to influence the complication rate and postsurgical hospitalization time.^45^

None of the remaining risk factors assessed in this investigation, including sex, neutering, breed, presurgical T4 concentration, thyroid lobe involvement, type of surgery, additional surgery performed, thyroid tumor size, local invasiveness, postsurgical levothyroxine calcitriol and calcium supplementation, and institution where surgery was performed, were associated with survival.

Unlike previous findings in dogs and humans,^7^, ^20^, ^46^, ^47^, ^48^ thyroid tumor size, bilateral involvement and local invasiveness did not influence outcome in our study. However, other investigations in affected dogs have highlighted that tumor diameter and volume, as well as bilateral thyroidectomy, had no effect on survival.^7^, ^18^, ^23^ Regarding local invasiveness, it is possible that the increasing attention of clinicians helped to achieve an earlier diagnosis and prevented disease progression in the affected dogs. Similarly to this series, dogs diagnosed with thyroid carcinoma undergoing thyroidectomy have an excellent outcome despite gross vascular invasion, with an MST of 621 days.^19^

The present investigation has some limitations. Because of its retrospective nature, some data were incomplete. There was also no standardized adjuvant chemotherapy protocol used to treat the affected dogs. The histopathological evaluation of the excised thyroid tumors was performed by different pathologists and samples could not be collected to review the diagnosis and add further testing.

In conclusion, dogs with thyroid tumors undergoing thyroidectomy have a long life expectancy. Undifferentiated carcinomas and metastases are major risk factors for thyroid cancer‐related death. Although metastases have a guarded outcome, extended survival is observed in certain dogs. Despite the rare presence of nonfollicular carcinoma and metastases, thyroidectomy should still be considered in some of these dogs. Similar to prior investigations, adjuvant chemotherapy did not prove to be beneficial in affected dogs. Unlike some studies, but confirming others, thyroid tumor size, invasiveness and bilateral thyroidectomy did not affect outcomes. The underlying reasons for thrombosis being a risk factor for death unrelated to thyroid cancer are unclear.

## CONFLICT OF INTEREST DECLARATION

Eric Zini serves as Associate Editor for the Journal of Veterinary Internal Medicine. He was not involved in review of this manuscript. No other authors have a conflict of interest.

## OFF‐LABEL ANTIMICROBIAL DECLARATION

Authors declare no off‐label use of antimicrobials.

## INSTITUTIONAL ANIMAL CARE AND USE COMMITTEE (IACUC) OR OTHER APPROVAL DECLARATION

Authors declare no IACUC or other approval was needed.

## HUMAN ETHICS APPROVAL DECLARATION

Authors declare human ethics approval was not needed for this study.
